# Pipecolic Acid, a Drought Stress Modulator, Boosts Chlorophyll Assimilation, Photosynthetic Performance, Redox Homeostasis, and Osmotic Adjustment of Drought-Affected *Hordeum vulgare* L. Seedlings

**DOI:** 10.3390/plants14131949

**Published:** 2025-06-25

**Authors:** Nagihan Aktas, Saad Farouk, Amal Ahmed Mohammed Al-Ghamdi, Ahmed S. Alenazi, Mona Abdulaziz Labeed AlMalki, Burcu Seckin Dinler

**Affiliations:** 1 Department of Biology, Faculty of Arts and Science, Sinop University, Sinop 57000, Turkey; naghan3714@gmail.com; 2 Agricultural Botany Department, Faculty of Agriculture, Mansoura University, Mansoura 35516, Egypt; 3 Department of Biological Sciences, Faculty of Science, King Abdulaziz University, P.O. Box 35009, Jeddah 21488, Saudi Arabia; aamghamdi@kau.edu.sa; 4 Department of Biological Sciences, College of Science, Northern Border University, Arar 91431, Saudi Arabia; ahmed.salem@nbu.edu.sa (A.S.A.); mona.almaliki@nbu.edu.sa (M.A.L.A.); 5 Center for Scientific Research and Entrepreneurship, Northern Border University, Arar 73213, Saudi Arabia

**Keywords:** chlorophyll metabolism, drought stress, *Hordeum vulgare*, oxidative homeostasis, pipecolic acid, systemic acquired resistance

## Abstract

While pipecolic acid (Pip) mediates morpho-physiological and molecular responses during biotic stress, its roles under drought remain an inexpressible mystery. The investigation aimed to elucidate the roles of a 30μM Pip pretreatment in alleviating drought injury on barley (*Hordeum vulgare* L. cv, Bülbül89) seedlings. Pip pretreatment under normal or drought conditions lowered the osmotic potential (Ψs) and water saturation deficit (WSD), while optimizing the relative water content (RWC), triggered osmotically energetic molecules (OEM) and salicylic acid (SA) accumulation, improving osmotic adjustment (OA), and boosting water retention and uptake capacity (WTC, and WUC), alongwith a considerable improvement in seedling growth over non-treated plants under such conditions. Additionally, Pip pretreatment improved chlorophyll (Chl), the chlorophyll stability index (CSI), pheophytin_a_, chlorophyllide_a_ (chlide_a_), chlorophyllide_b_ (chlide_b_), chl_a_/chlide_a_, chl_b_/chlide_b_, protoporphyrin, Mg-protoporphyrin, protochlorophyllide, and photosynthetic performance over non-treated plants under such conditions. Pip pretreatment preserves redox homeostasis in drought-stressed plants by accumulating antioxidant solutes alongside the activation of superoxide dismutase and glutathione reductase over non-treated plants. Drought distinctly reduced Ψs (more negative), RWC, photosynthetic pigment, CSI, chlorophyll assimilation intermediate, and photosynthetic performance, with an increment in chlorophyll degradation intermediate and nonenzymatic antioxidant solutes. Drought maintains OA capacity via a hyper-accumulation of OEM and SA, which results in higher WSD, WTC, and WUC. Drought triggered an oxidative burst, which was associated with a decline in the membrane stability index. These findings highlight Pip’s capability for lessening drought stress-induced restriction in barley seedlings via bolstering oxidative homeostasis, OA capacity, and stabilizing chlorophyll biosynthesis. Future research must elucidate the precise molecular mechanisms underlying Pip’s action in alleviating drought injury.

## 1. Introduction

Climate change and human activities pose numerous obstacles to agricultural sustainability and food security, owing to rising temperatures, prolonged dry spells, inconsistent rainfall patterns, and heightened needs for water and energy [1]. Drought is the foremost abiotic stress in arid and semi-arid districts that threatens crop productivity [2]. Over the last decade, drought-related crop production economic losses have risen to roughly USD 30–44 billion worldwide, and freshwater supplies are projected to drop by 50%. In contrast, agricultural water requirements will increase twofold by 2050 [3].

Drought severely impedes morpho-anatomical, physio-biochemical, and molecular features, which ultimately hampers plant growth and productivity, reduces relative water content, diminishes membrane stability, and alters photosynthetic performance [1,4]. Moreover, drought-induced oxidative bursts in various plants cause a disruption of normal metabolic pathways, including lipid peroxidation, upsetting cell membrane integrity, protein and nucleic acid destruction, the deformation of cellular organelles, and eventually, inducing cell and whole-plant death [5].

Photosynthesis represents the most vital plant metabolic process and is greatly affected by environmental obstacles, arising from photochemical restrictions [1,6]. Photosynthesis in drought-affected plants is directly restricted through the dysfunction of ribulose 1,5-bisphosphate carboxylase oxygenase (Rubisco) and carbonic anhydrase (CA) activities, alongside a malformation of chloroplasts and chlorophyll degradation [6,7]. Such declines are linked to reduced chlorophyll (Chl) assimilation, photosynthetic rate, and photoassimilate production [1,5]. Chlorophyll content relies on the dynamic balance between the assimilation and catabolism pathways. Chlorophyll metabolism is a multifaceted enzymatic process that involves various genes and is affected by environmental stress [8,9]. Recently, there has been a limited amount of research on Chl metabolism in drought conditions, and further clarification is needed.

The ability of plants to endure water scarcity relies on several metabolic pathways that regulate plant–water relations and preserve ion homeostasis [1,7]. The key pathways include the hyper-accumulation of osmotically energetic molecules (OEM), i.e., total free amino acids (TAA), proline (Pro), and soluble sugar (SS) [6]. Genotypes with elevated osmotic adjustment (OA) are capable of maintaining consistently higher osmotic potential (Ψs), supporting turgor maintenance, preserving root development, and lessening the destructive effects of drought [5,10]. Although OA is accepted as a crucial component of drought tolerance strategies in various cultivars, its importance for barley’s drought resilience remains uncertain. Plants accumulate significant inorganic ions and synthesize OEM to ensure steady water absorption and sufficient pressure potential, which is crucial for growth and the maintenance of photosynthetic capacity [1,2]. The prevalent OEMs under drought stress are amino or imino acids (glutamate, Pro, pipecolic acid ‘Pip’, andectoine), soluble carbohydrates, and methylated onium compounds [10]. They play a vital role in stressed plants as osmoprotectants, nitrogen storage, modulation of cellular pH, and elimination of free radicals [11]. Conversely, these endogenous anti-drought accumulations cannot sufficiently support crop growth and productivity under extended drought, highlighting the need to apply external agents to induce drought resilience.

Various approaches have been developed to lessen drought injury, i.e., developing drought-tolerant genotypes (via traditional breeding or genetic engineering). Nevertheless, plants are not fully protected from stress mutilation due to their restricted genetic self-defense aptitude. This clarifies why plant scientists are exploring alternatives to improve plants’ ability to withstand harsh environmental conditions using innovative materials, including Pip [12,13]. The non-protein heterocyclic amino acid L-Pip, known as homoproline, serves as an intermediate in the catabolism of D, L-lysine [14]. Pip is often assimilated in all biota (microorganisms, animals, and plants) and acts as a precursor for naturally bioactive molecules, potentially contributing to the progress of the systemic acquired resistance ‘SAR’ response [14]. L-Pip and L-proline possess similar chemical structures, differing by only one carbon atom in their ring [15]. L-Pip has been identified as a compatible solute for several microorganisms and non-halophilic plants [15,16]. Previous studies have shown that Pip can trigger pathogen resistance in tomatoes via an oxidative homeostasis strategy [14]. Pip occupies multiple roles in different biotic stresses, but its roles in abiotic stresses, mainly drought, remain largely ambiguous. In this context, Kucukkalyon and Seckin Dinler [13] provide the first proof that Pip can induce salinity tolerance by minimizing the oxidative damage associated with an improved chlorophyll concentration and seedling growth. Recently, a few studies have suggested that plants can accumulate Pip under abiotic stress [17]. Pip is produced from N-hydroxy-pipecolic (NHP) by two enzymatic pathways that activate aminotransferase (ALD1) and flavin-dependent monooxygenase1 (FMO1) in the cytosol [18]. Gene expression profiling data suggest that drought significantly alters the activities of ALD1 and FMO1, thus inducing drought tolerance [19]. Hence, Pip’s involvement in plant drought tolerance requires further exploration [20]. Nonetheless, advanced studies are required to understand Pip’s function in plants’ drought tolerance, as well as its connection to photosystems and antioxidant systems.

Barley (*Hordeum vulgare* subsp. *vulgare* L., family Poaceae) comes in the fourth most important cereal cultivated worldwide for food, fodder, fermentation, and certain industries, which is cultivated on about 47.3 million hectares, with 151.9 million tons produced in 2022/2023 [21]. Globally, barley consistently faces water shortages, leading to a decline in cultivated areas and crop production [22]. Hence, it is crucial to boost barley production to satisfy the food requirements of the overgrowing population or even enhance productivity in challenging climate changes [23]. Nonetheless, to the best of our knowledge, no study has appraised the Pip’s specific roles in improving plants, i.e., barley’s tolerance to drought conditions, and the pretreatment of Pip under abiotic stress is unprecedented, while its role in eliciting abiotic stress factors remains mysterious and requires further exploration. So, this study sought to clarify the effectiveness and regulatory roles of Pip treatment in improving barley’s drought tolerance, focusing on changes in growth and anatomical, physiological, and biochemical features. It was hypothesized that Pip pretreatment attenuates the undesirable impacts of drought on barley plants, as it can improve osmotic adjustment capacity, chlorophyll assimilation, and photosynthesis efficiency and bolster antioxidant defense systems. These findings offer a novel water-conserving method for arid and semi-arid areas and ensure secure barley productivity, particularly from the perspective of climatic changes.

## 2. Results

An analysis of variance of the current study showed that Pip pretreatment under normal or drought conditions significantly affected all morpho-physiological and anatomical features of barley seedlings (Supplementary Material).

### 2.1. Vegetative Growth

**Figure 1:**
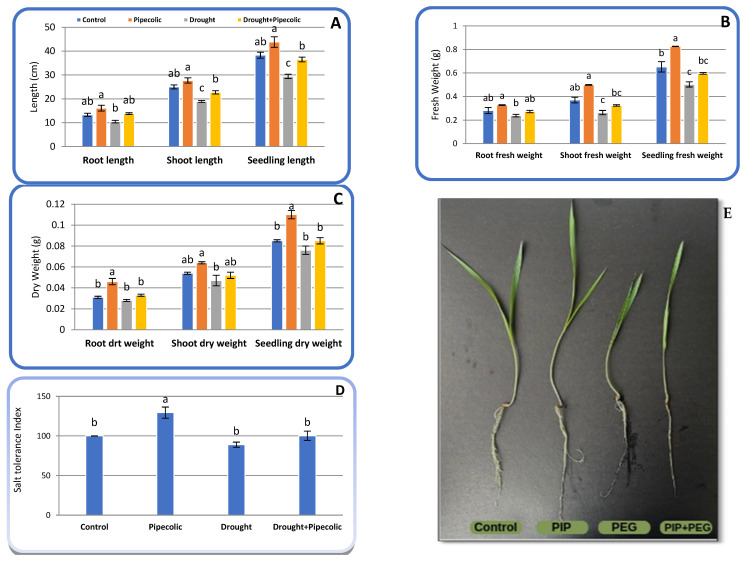
Effect of drought stress and pipecolic acid and their interaction on barley seedling growth attributes and tolerance index. Data represent the mean ± standard error (*n* = 5). Significant differences between groups were tested by one-way analysis of variance (ANOVA, *p* ≤ 0.05). Data of each column indicated by the same letters are not significantly different (*p* ≤ 0.05) following Tukey’s test. (**A**) Root, shoot, and seedling length (cm); (**B**) root, shoot, and seedling fresh weight (g); (**C**) root, shoot, and seedling dry weight (g); (**D**) salt tolerance index; (**E**) seedling features under treatment; Pip, pipecolic acid; PEG, polyethylene glycol.

Seedling growth attributes including shoot length (SL), root length (RL), seedling length (SeL), shoot fresh weight (SFW), root fresh weight (RFW), seedling fresh weight (SeFW), shoot dry weight (SDW), root dry weight (RDW), seedling dry weight (SeDW), and drought tolerance index (TI) were significantly (*p* ≤ 0.05) affected by drought, Pip, and their interaction compared to untreated control plants (Figure 1A–E). Figure 1A–D indicates that drought stress significantly (*p* ≤ 0.05) reduced SL, SeL, SFW, and SeFW by 24.40, 23.23, 28.64, and 22.85%, respectively, while also causing a non-significant decrease in RL, RFW, SDW, RDW, SeDW, and TI by 21.80, 15.30, 12.96, 9.67, 10.58, and 12.0%, respectively, compared to untreated control plants. Pre-treatment with Pip under control conditions noticeably improved barley seedling growth, proportional to untreated control plants. Pip pretreatment significantly (*p* ≤ 0.05) increased SeFW (26.68%), RDW (48.38%), SeDW (29.41%), and TI (29.40%), along with a non-significant increment in SL (10.80%), RL (21.05%), SeL (14.36%), SFW (34.59%), RFW (16.72%), and SDW (18.51%) relative to untreated control plants. Pre-treatment with Pip under drought stress significantly (*p* ≤ 0.05) abolished to some extent the injury of drought on barley’s growth features and provided values closest to those of the control plants. As compared with drought-affected plants, pre-treatment with Pip significantly (*p* ≤ 0.05) enhanced SL (20.10%) and SeL (24.14%), while also showing a non-significant increase in RL (32.69%), SFW (22.72%), RFW (14.70%), SeFW (18.68%), SDW (10.63%), RDW (17.85%), SeDW (11.84%), and TI (12.64%).

### 2.2. Anatomical Study and Stomatal Number

**Figure 2:**
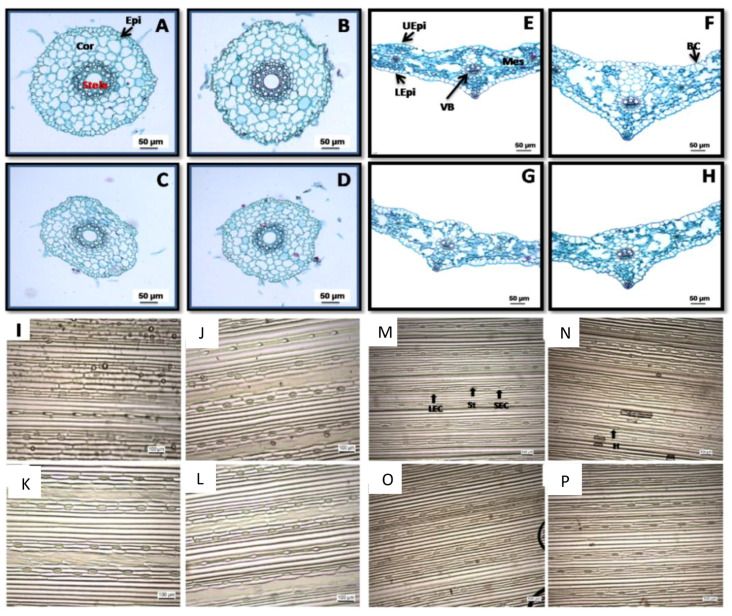
Anatomical structure of root (**A**–**D**), leaf (**E**–**H**), and the stomatal density in adaxial (**I**–**L**) and abaxial (**M**–**P**) epidermis of barley and seedling as affected by drought and pipecolic acid ((**A**,**E**,**I**,**M**) = control; (**B**,**F**,**J**,**N**) = 30 μm pipecolic acid; (**C**,**G**,**K**,**O**) = drought; (**D**,**H**,**L**,**P**) = drought + pipecolic acid). BC, Bulliform cells; Cor, cortex; Epi, epidermis; H, hair; LEC, long epidermal cell; LEpi, lower epidermis; Mes, mesophyll tissue; SEC, short epidermal cell; ST, stomata; UEpi, upper epidermis; VB, vascular bundle).

Table 1 and Figure 2A–D show that drought stress negatively affects root anatomical features. The reduction values were measured in the thickness (µm) of epidermis and cortex, stele diameter, and vascular tissue thickness of the barley root by 66.66, 38.69, 38.83, and 43.5%, respectively, over the control treatment. Cross-sections of barley roots exhibited notable changes in root anatomical features induced by Pip under normal conditions, which increased all anatomical features of the root by 192.7% (cortex thickness), 14.32% (stele diameter), and 23.90% (thickness of vascular tissue) over the nontreated plants. Pip pretreatment under drought markedly increased root anatomical features compared with untreated drought-affected plants.

The results in Table 1 and Figure 2E–H also showed that drought stress negatively affects the majority of leaf anatomical features. Drought stress significantly (*p* ≤ 0.05) decreased the leaf blade thickness, mesophyll tissue thickness, and thickness of the leaf at the midrib region, main vascular bundle width, and xylem vessel diameter more than the control, by 3.34, 10.52, 6.25, 14.28, and 20.41%. Cross-sections of the barley leaves exhibited notable alterations in the leaf anatomical features induced by Pip. In this investigation, Pip treatment enlarged the thickness of the leaf blade, which is attributed to the rise in the thickness of mesophyll tissue. Furthermore, the thickness of the leaf blade in the midrib region was likewise elevated, which is attributable to the rise in the midrib vascular bundle dimension, coupled with the radial diameter of the metaxylem vessel. Pip pretreatment under drought markedly enhanced most of the leaf’s anatomical features in comparison to the untreated drought-affected plants, with increases of 18.18, 23.52, 21.87, and 10.52% for leaf blade thickness, mesophyll tissue thickness, midrib region thickness, and xylem vessel diameter, respectively. In comparison to the control treatment, Pip treatment relieves the injurious effects of drought on leaf anatomy by increasing the leaf succulence and vascular tissue.

The stomatal density varied greatly among the treatments (Table 1; Figure 2I–P) of drought and/or Pip pretreatment. Table 1 and Figure 2I–P reveal that drought stress and/or Pip pretreatment under drought raised the number of stomata on both the adaxial and abaxial surfaces over the control. Furthermore, the results suggest that, under the control condition, the pretreatment of Pip decreased the stomatal density over the nontreated control plants (Figure 2I–P). Moreover, the micrographs of the epidermal cells illustrated that alterations in cell size were associated with the decline in leaf area.

### 2.3. Chlorophyll Metabolic Pathways Intermediates

**Figure 3:**
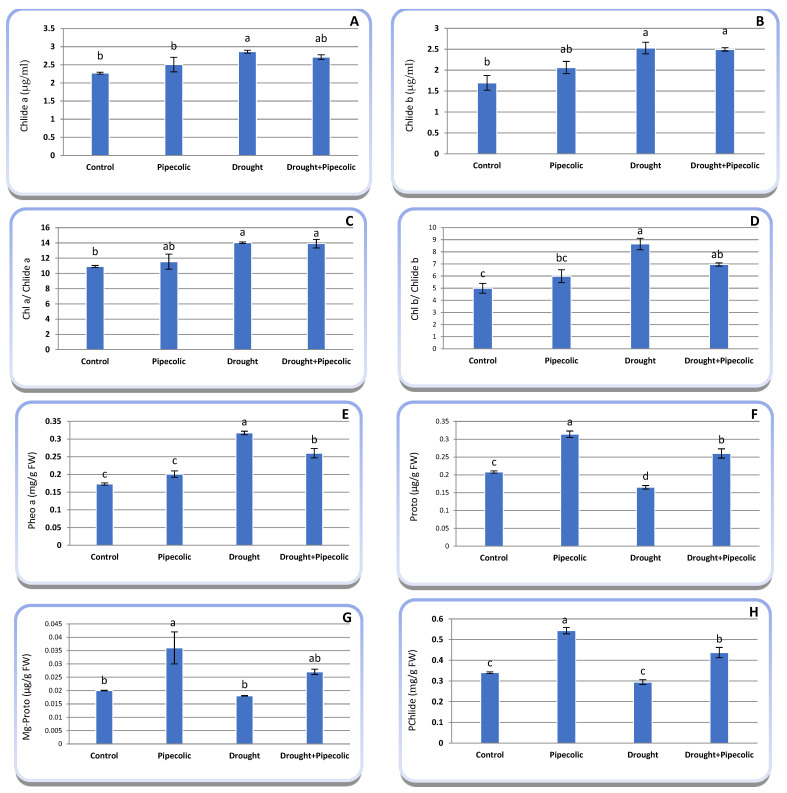
Effect of drought stress and pipecolic acid and their interaction on chlorophyll assimilation and degradation intermediates of barley seedlings. Data represent the mean ± standard error (*n* = 5). Significant differences between groups were tested by one-way analysis of variance (ANOVA, *p* ≤ 0.05). Data of each column indicated by the same letters are not significantly different (*p* ≤ 0.05) following Tukey’s test. (**A**), Chlorophyllide_a_ (Chlide_a_); (**B**), Chlorophyllide_b_ (Chlide_b_); (**C**), Chlorophyll_a_ (Chl_a_)/Chlide_a_; (**D**), Chlorophyll_b_ (Chl_b_)/Chlide_b_; (**E**), Pheophytin_a_ (Pheo_a_); (**F**), protoporphyrin (Proto); (**G**), magnesium–protoporphyrin (Mg-Proto); (**H**), Protochlorophyllide (PChlide).

Evaluating the chlorophyll metabolic pathways intermediates (biosynthesis and catabolism) can offer valuable insights into understanding the responses of chlorophyll metabolism to drought and/or Pip pretreatment. The data in Figure 3A–H indicate that the chlorophyll metabolic pathway intermediates were severely altered in barley seedlings by drought and/or Pip pretreatment. Drought stress significantly (*p* ≤ 0.05) increased the chlorophyll catabolism intermediates (Chlide_a_, Chlide_b_, and Pheo_a_), as well as the ratios of Chl_a_/Chlide_a_ andChl_b_/Chlide_b_ relative to the control plants. The raised in Chlid_a_, Chlide_b_, Chl_a_/Chlide_a_, and Chl_b_/Chlide_b_Pheo_a_ reached 26.03, 49.17, 28.51, 73.30, and 83.23%, respectively, under drought correspondingly over the control plants. Conversely, drought stress significantly (*p* ≤ 0.05) lowered Proto (20.67%), with a non-significant reduction in Mg-Proto (10.00%) and Pchlide (13.52%) compared to the control plants.

Pretreatment with Pip under control conditions significantly (*p* ≤ 0.05) elevates the chlorophyll biosynthesis intermediate concentrations over non-treated plants. In this regard, Pip pretreatment under normal conditions raised Proto, Mg-Proto, and Pchlide by 50.96, 80, and 59.70%, respectively, over control plants. Conversely, Pip pretreatment on drought-affected seedlings significantly (*p* ≤ 0.05) boosted Proto (57.57%) and Pchlide (48.63%), along with a non-significant rise in Mg-Proto (50.00%) compared to the untreated drought-affected plants.

Additionally, Pip pretreatment under normal conditions did not significantly raise Chlide_a_, Chlide_b_, Chl_a_/Chlide_a_, Chl_b_/Chlide_b_, and Pheo_a_ when compared to the untreated control plants. Also, Pip pretreatment of drought-stressed seedlings resulted in a non-significant reduction in Chlide_a_, Chlide_b_, Chl_a_/Chlide_a_, and Chl_b_/Chlide_b_, while there was a significant decline in Pheo_a_ compared to the untreated drought-stressed seedlings.

### 2.4. Photosynthetic Pigments

**Figure 4:**
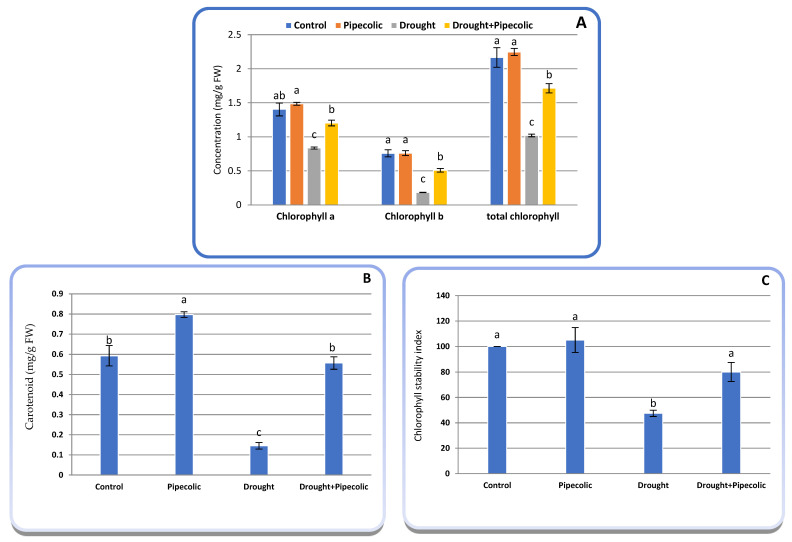
Effect of drought stress and pipecolic acid and their interaction on photosynthetic pigment concentration, and chlorophyll stability index of barley seedlings. Data represent the mean ± standard error (*n* = 5). Significant differences between groups were tested by one-way analysis of variance (ANOVA, *p* ≤ 0.05). Data of each column indicated by the same letters are not significantly different (*p* ≤ 0.05) following Tukey’s test. (**A**) chlorophyll_a_, chlorophyll_b_, and total chlorophyll concentration; (**B**) carotenoid concentration; (**C**) chlorophyll stability index.

Figure 4A–C revealed that the concentrations of chlorophyll (a,b, and total), total carotenoids, and chlorophyll stability index were severely altered by drought and/or Pip pretreatment. Drought caused a significant (*p* ≤ 0.05) decline in chlorophyll_a_ (40.58%), chlorophyll_b_ (75.72%), total chlorophyll (82.86%), total carotenoid (75.54%), and the chlorophyll stability index (47.47%) relative to control plants (Figure 4A–C). Pretreatment with Pip to non-stressed plants led to a non-significant increase in Chlorophyll_a_, chlorophyll_b_, total chlorophyll, and the chlorophyll stability index, along with a significant rise in the carotenoid concentration proportionate to the untreated seedlings, with increases of 5.54, 0.33, 3.73, 105, and 34.40% (Figure 4A–C). Pip pretreatment on drought-affected plants proscribed the drought-related photosynthetic pigment deficiency and chlorophyll stability index by sustaining a greater concentration in leaves relative to the untreated drought-exposed plants.

### 2.5. Photosynthetic Performance

**Figure 5:**
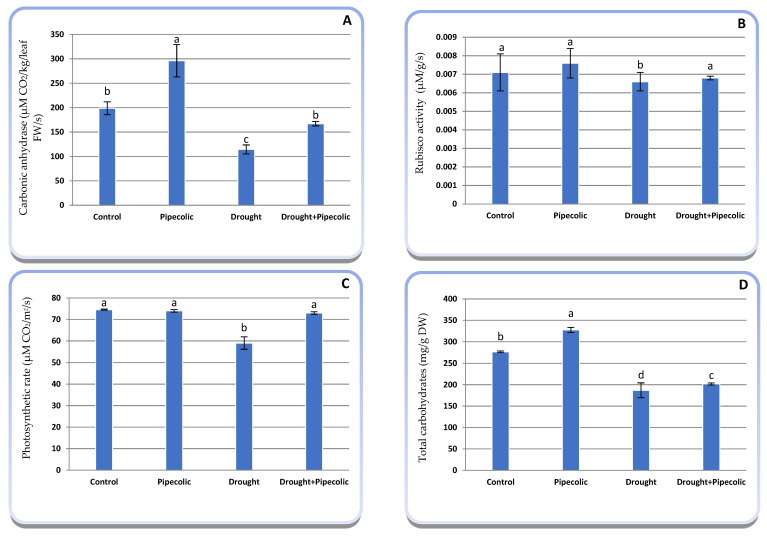
Effect of drought stress and pipecolic acid and their interaction on photosynthetic efficiency of barley seedlings. Data represent the mean ± standard error (*n* = 5). Significant differences between groups were tested by one-way analysis of variance (ANOVA, *p* ≤ 0.05). Data of each column indicated by the same letters are not significantly different (*p* ≤ 0.05) following Tukey’s test. (**A**) carbonic anhydrase activity; (**B**) Rubisco activity; (**C**) photosynthetic rate; (**D**) total carbohydrates.

Data presented in Figure 5A–D show that the photosynthetic performance attributes (CA, Rubisco, Pn, and total carbohydrates) significantly (*p* ≤ 0.05) declined under drought stress compared to control plants. Alternatively, Pip pretreatment under normal conditions significantly (*p* ≤ 0.05) raised the CA (48.97%) and total carbohydrates (15.54%), along with a nonsignificant rise in Rubisco (7.04%), while maintaining a steady value for Pn over the nontreated plants. The same figure showed that Pip pretreatment to drought-affected plants in most cases mitigated the harmful effects of drought on the photosynthetic attributes over the non-treated drought-affected plants, which significantly increased CA, Rubisco, Pn, and total carbohydrates by 46.15, 3.033, 23.72, and 7.96%, respectively.

### 2.6. Osmotically Energetic Molecules

**Figure 6:**
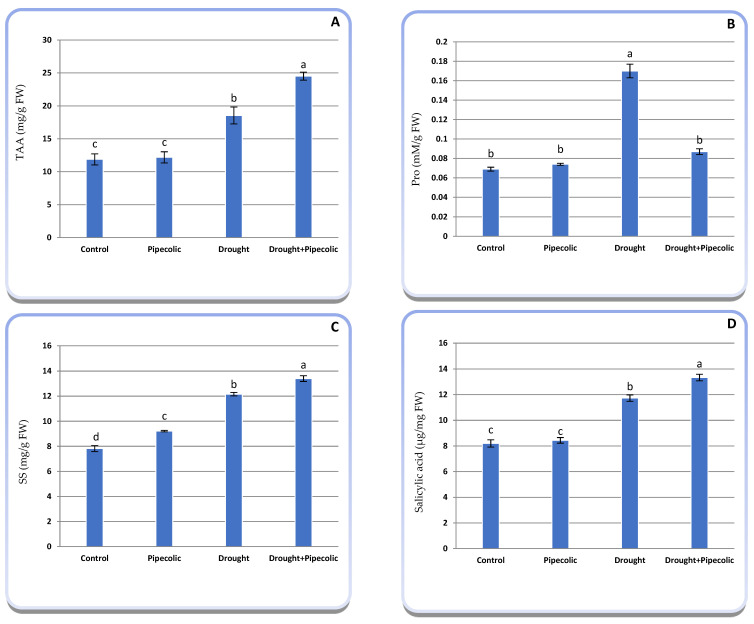
Effect of drought stress and pipecolic acid and their interaction on OAM and salicylic acid concentration of barley seedlings. Data represent the mean ± standard error (*n* = 5). Significant differences between groups were tested by one-way analysis of variance (ANOVA, *p* ≤ 0.05). Data of each column indicated by the same letters are not significantly different (*p* ≤ 0.05) following Tukey’s test. (**A**) total amino acid concentration; (**B**) proline concentration; (**C**) soluble sugar concentration; (**D**) salicylic acid concentration. TAA, total amino acid; Pro, proline; SS, soluble sugars.

Important variations were identified for the drought and Pip treatments regarding OEM and SA concentrations in barley seedlings (Figure 6A–D). Drought stress significantly (*p* ≤ 0.05) increased TAA (56.19%), Pro (146.37%), and SS (55.51%) in barley seedlings relative to the control plants. Pretreatment with Pip under normal conditions induces a non-significant increase in TAA and Pro, meanwhile significantly (*p* ≤ 0.05) increasing SS (17.80%) relative to the nontreated control plants. Alternatively, under drought conditions, the Pip pretreatment significantly (*p* ≤0.05) increased TAA and SS by 106.48% and 71.37%, respectively, which is associated with a nonsignificant increase in the Pro concentration in barley seedlings over the nontreated control plants. The figure also indicates that the SA concentration in barley seedlings was significantly (*p* ≤ 0.05) elevated by drought and/or Pip pretreatment over the non-treated control plants. The greatest concentration of SA was observed in the drought-affected plants that were pretreated with Pip, attaining 62.40% over the control plants.

### 2.7. Water Status and Osmotic Adjustment

**Figure 7:**
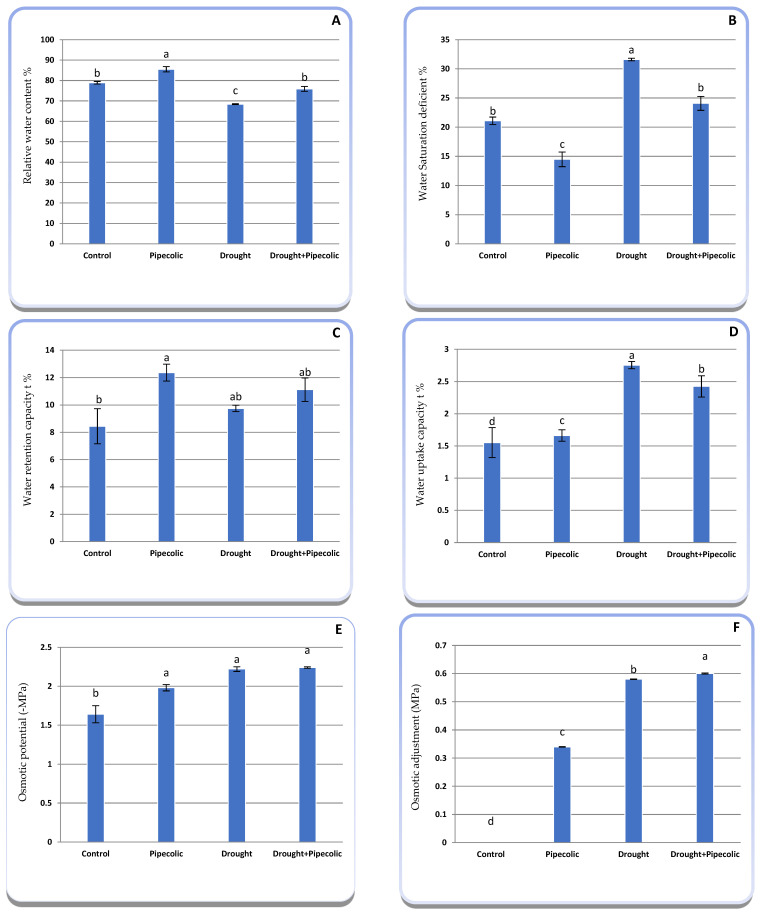
Effect of drought stress and pipecolic acid and their interaction on leaf water status and osmotic adjustment capacity of barley seedlings. Data represent the mean ± standard error (n = 5). Significant differences between groups were tested by one-way analysis of variance (ANOVA, *p* ≤ 0.05). Data of each column indicated by the same letters are not significantly different (*p* ≤ 0.05) following Tukey’s test. (**A**) relative water content %; (**B**) water saturation deficient%; (**C**) water retention capacity%; (**D**) water uptake capacity%; (**E**) osmotic potential; (**F**) osmotic adjustment.

Drought stress significantly reduced RWC (13.32%) and Ψs (more negative by 35.36%), while leading to a significant (*p* ≤ 0.05) rise in WSD (49.83%), WUC (77.46%), and OA capacity (35.36%), along with a non-significant increase in WTC (15.39%) over the well-watered seedlings (Figure 7A–F). Under normal conditions, Pip pre-treatment significantly (*p* ≤ 0.05) increased RWC, WTC, WUC, and OA capacity by 8.37, 46.39, 5.15, and 20.73%, respectively, while decreasing WSD (31.34%) and Ψs (more negative 34.00%). Under drought conditions, Pip pre-treatment significantly (*p* ≤ 0.05) increased the RWC and OA capacity, while decreasing WSD, Ψs (more negative), and WUC, along with a nonsignificant increase in WTC relative to the untreated drought-affected plants, although all trials surpassed those of the nontreated well-watered control plants.

### 2.8. Oxidative Biomarkers

**Figure 8:**
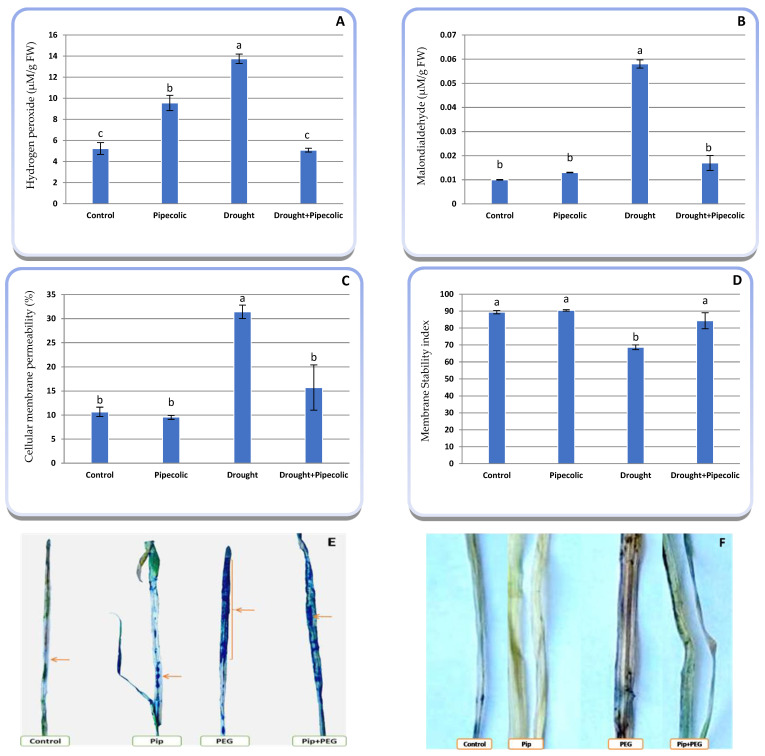
Effect of drought stress and pipecolic acid and their interaction on oxidative biomarkers and in situ localization of superoxide and hydrogen peroxide by nitro blue tetrazolium staining and DAB staining of barley seedlings. Data represent the mean ± standard error (*n* = 5). Significant differences between groups were tested by one-way analysis of variance (ANOVA, *p* ≤ 0.05). Data of each column indicated by the same letters are not significantly different (*p* ≤ 0.05) following Tukey’s test. (**A**) hydrogen peroxide concentration; (**B**) malondialdehyde concentration; (**C**) cellular membrane permeability percentage; (**D**) membrane stability index; (**E**) in situ localization of superoxide anion; the arrow indicate the blue color of superoxide localization (**F**) in situ localization of hydrogen peroxide indicated in brown color. Pip, pipecolic acid; PEG, polyethylene glycol 6000.

To determine the role of Pip in alleviating the effects of drought-induced oxidative damage on barley seedlings, H_2_O_2_, MDA, CMP, and MSI, in addition to the histochemistry of superoxide and H_2_O_2_, were studied (Figure 8A–F). Drought stress significantly (*p* ≤ 0.05) induced a substantial accumulation of H_2_O_2_ (162.71%) concentration in barley seedlings compared to the control plants. The massive accumulation of H_2_O_2_ can trigger several biochemical non-specific oxidations of lipids, leading to a 480% increase in the MDA concentration relative to the control plants. This oxidation process can also be shown through dysfunction of the cell membrane, expressed by a rise in CMP (194.65%) associated with a 23.22% reduction in MSI (Figure 8A–F).

The same Figure indicates that pre-treatment with Pip was effective in lowering drought-induced oxidative biomarkers compared with non-treated plants. Regarding non-treated drought-affected plants, Pip pretreatment had surprisingly declined the assembly of H_2_O_2_, MDA buildup, and CMP by 63.07, 70.68, and 49.95%, respectively, meanwhile increasing MSI by 22.86%. The same figure shows that, under normal conditions (without drought), Pip pretreatment improved MSI; while a nonsignificant decrease in the percentage of CMP was associated with a small increase in H_2_O_2_ and MDA concentrations.

Histochemical staining using DAB and NBT revealed the buildup of leaves’ H_2_O_2_ and O_2_^−^, respectively. Nitro-blue tetrazolium dye reacts with endogenous O_2_^−^, resulting in the development of a blue formazan compound, which signifies the localization of O_2_^−^ anions. The strength of blue stains was noted to be minimal in control and Pip-pretreated plants, whilst it was significantly elevated for drought-treated plants (Figure 8E). Pretreated drought-affected plants with Pip exhibited a further decline in blue stain strength compared to the non-treated drought-affected plants. Likewise, histochemical staining with DAB visualizes H_2_O_2_ levels by showing brown spots. The tiny strength of brown spots was visible in the control and Pip-pretreated plants, while the drought-exposed plants showed a superior strength of brown spot development (Figure 8F). Under drought, a lesser brown stain in those plants pretreated with Pip was observed compared to the drought-affected plants only. These outcomes were established in connection with quantified concentrations of H_2_O_2_ in the plant.

### 2.9. Non-Enzymatic Antioxidant Metabolites

**Figure 9:**
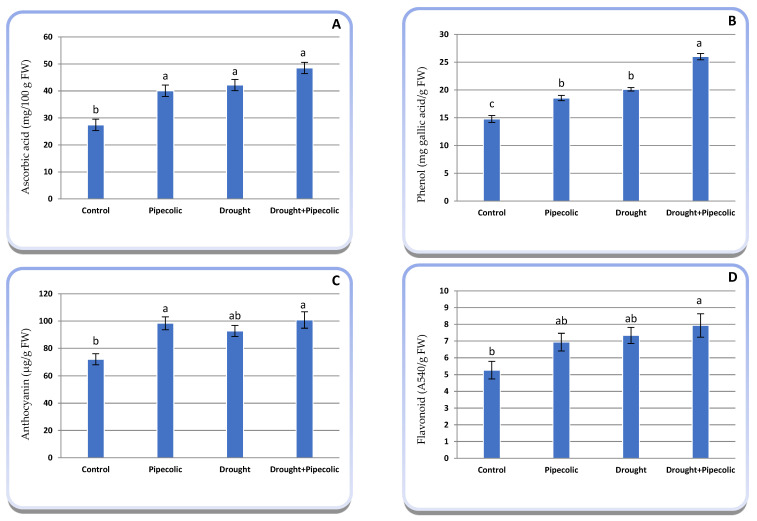
Effect of drought stress and pipecolic acid and their interaction on non-enzymatic antioxidant solutes of barley seedlings. Data represent the mean ± standard error (*n* = 5). Significant differences between groups were tested by one-way analysis of variance (ANOVA, *p* ≤ 0.05). Data of each column indicated by the same letters are not significantly different (*p* ≤ 0.05) following Tukey’s test. (**A**) ascorbic acid concentration; (**B**) phenol concentration; (**C**) anthocyanin concentration; (**D**) flavonoid concentration.

Figure 9A–D proves that drought significantly raised the concentrations of ascorbic acid and phenols, while showing a non-significant increase in anthocyanin and flavonoid concentrations in barley seedlings relative to the control. Elevated antioxidant solutes were observed in Pip-treated plants under normal or drought-stressed conditions relative to untreated seedlings under such conditions, confirming the idea that Pip exhibits noteworthy antioxidant activity, either directly or indirectly, by triggering the accumulation of antioxidants (Figure 9A–D). The highest levels of ascorbic acid, phenols, anthocyanins, and flavonoids were achieved through applying Pip under drought condition, resulting in increases in ascorbic acid (76.95%), phenols (75.84%), anthocyanins (40.00%), and flavonoids (50.64%), compared to the untreated control plants (Figure 9A–D).

### 2.10. Antioxidant Enzymes

**Figure 10:**
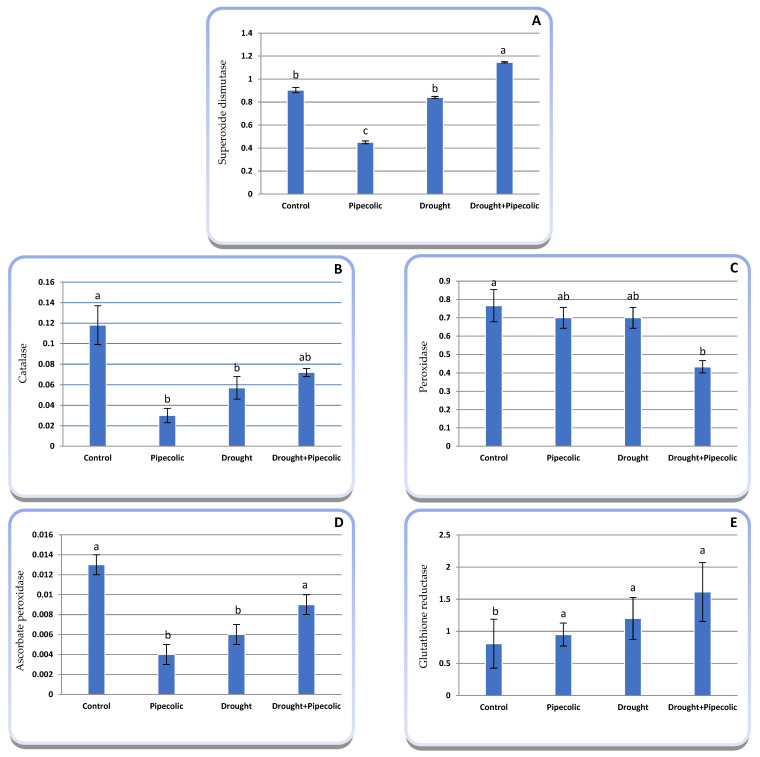
Effect of drought stress and pipecolic acid and their interaction on antioxidant enzymatic activities (U/mg protein) of barley seedlings. Data represent the mean ± standard error (*n* = 5). Significant differences between groups were tested by one-way analysis of variance (ANOVA, *p* ≤ 0.05). Data of each column indicated by the same letters are not significantly different (*p* ≤ 0.05) following Tukey’s test. (**A**) superoxide dismutase activity; (**B**) catalase activity; (**C**) peroxidase activity; (**D**) ascorbate peroxidase activity; (**E**) glutathione reductase activity.

The activities of SOD, CAT, POX, APX, and GR were evaluated to verify the protective role of Pip on oxidative detoxification in drought-stressed barley seedlings. As shown in Figure 10A–E, the activities of SOD, and POX in drought-affected seedlings non-significantly declined by 6.96%, and 8.61%, respectively, over the control plants, Meanwhile, CAT and APX activities in drought-affected seedlings significantly decreased by 51.69%, and 53.84%, respectively, over the control plants. Alternatively, GR activity significantly increased by 48.88% under drought, relative to the control plants.

Pip pre-treatment under the control condition significantly reduced the activities of SOD (50.22%), CAT (74.57%), and APX (69.23%) alongside a non-significant decline in POX (8.61%), while significantly enhancing GR (17.74%) activity relative to the untreated control plants. Pre-treatment with Pip to drought-affected seedlings significantly boosted SOD and GR by 26.76 and 100% over the control plants and by 40.66 and 34.33%, respectively, over the nontreated drought-affected plants. Meanwhile, Pip pretreatment under drought led to a nonsignificant increase in CAT (26.31%) and a significant improvement in APX (50.00%) activity, whereas POX activity experienced a non-significant decline of 38.14% over the untreated drought-affected seedlings.

## 3. Discussion

Growth suppression is a critical sign of drought injury, caused by numerous physio-biochemicals, anatomical, and molecular modifications [1,5,6]. The ongoing investigation noted that drought-affected barley seedlings were shorter and exhibited lower biomass associated with a decline in tolerance index (Figure 1D). Plant growth relies on several processes linked to cell division and differentiation in meristematic regions, along with cell expansion in the elongation zone, which is adversely impacted by water and osmotic and turgor potentials during drought [5]. The current study and earlier references indicate that the impact of drought on plant growth may result from a decline in water potential and cell division/expansion caused by losses in turgor, leaf RWC, and Ψs (Figure 7) [5]. Drought similarly hinders plant growth via diminishing photosynthesis and restricting leaves’ CO_2_ uptake, which in turn, lowers photosynthetic rates and chlorophyll degradation (Figure 3, Figure 4 and Figure 5) [1]. Moreover, drought stress injury is chiefly coupled with the excessive accumulation of reactive oxygen species “ROS” (Figure 8) that triggers oxidative bursts, resulting in the deterioration of vital cellular components, disrupting the plant’s water status (Figure 7) and hindering electron transport pathways, resulting in diminished photosynthetic efficiency and lower availability of the photoassimilate for the activity-growing organs [5,6]. Regularly, Pip consistently plays a decisive role in raising the growth of drought-affected plants by modifying various metabolic pathways. The role of Pip in mitigating abiotic-affected plants stays unclear, and so far, no definite receptors have been recognized. Pip pretreatment may boost drought tolerance, as suggested in this study through hyperaccumulation of OEM (Figure 6), alongside enhancing water uptake capacity (Figure 7), sustaining elevated RWC and WTC, and ultimately high OA capacity (Figure 7) along with improving root anatomy (Table 1; Figure 2), which is essential for cell division, elongation, and ultimately, plant growth. Also, Pip pretreatment boosted photosynthetic pigments (Figure 3 and Figure 4) and activated both Rubisco and CA enzymes (Figure 5), along with improving and maintaining a high photosynthetic rate and photoassimilate production (Figure 5D), which are vital for plant growth and development. Additionally, the Pip pretreatment might be intensifying non-enzymatic antioxidant solutes (Figure 9) within plant cells, while simultaneously declining in oxidative biomarkers (Figure 8). Another explanation for the enhancement of barley seedling growth by Pip pretreatment may be a result of hyperaccumulation of ascorbic acid (Figure 9), which is essential for modifying the cell cycle, encouraging quiescent cells into division, and speeding up cell expansion and elongation [24]. The present study suggests that Pip pretreatment might mitigate the injury of drought on barley seedlings.

Chlorophyll plays a critical role in photosynthesis and biomass gathering. Thus, any disruption in its metabolism (assimilation and catabolism) and functionality reduces the effectiveness of light-induced excitation energy conveyance and, ultimately, affects CO_2_ assimilation [9]. In this study, chlorophyll severely declined with drought. Nonetheless, Pip pretreatment enhanced their levels (Figure 4). Chlorophyll deprivation and leaf yellowing in drought-affected plants, as indicated in this study and previously validated, is an outstandingly established occurrence [1]. It was suggested that the effect of drought on chlorophyll concentration could be linked to its influence on their biosynthetic pathways (Figure 3) [8]. Based on the current study, the decline in chlorophyll under drought probably resulted from a decrease in porphyrin compound synthesis and heightened chlorophyll breakdown (Figure 3). The chlorophyll degradation pathway is a preserved multistep enzymatic process. A natural catabolite of chlorophyll is Pheo_a_, which is generated when Mg is removed from the tetrapyrrole ring [9,25]. Another chlorophyll decomposition product is Chlide, which is formed by chlorophyllase via losses of their phytol tails [26]. There is limited information regarding the effect of environmental stressors on chlorophyll metabolic intermediates in different plants [8,9]. The injury of drought on chlorophyll is chiefly associated with a massive accumulation of ROS (Figure 8) coupled with an accelerating clustering and dysfunction of chloroplasts [7]. Examining the metabolic intermediates involved in the synthesis (Proto IX, Mg-Proto IX, and Pchlide) or degradation (Pheo_a_, Chlide_a_, andChlide_b_) pathways of chlorophyll can significantly reveal the mechanism through which drought and Pip affect chlorophyll concentration. The current study suggested that Pip modulates chlorophyll levels under drought by reducing chlorophyll degradation and increasing its bioassimilate intermediates (Figure 3), along with boosting Rubisco activity (Figure 5B) over nontreated drought-affected plants. It is, thus, plausible that the functionality of the photosynthetic system was sustained more efficiently in the Pip-treated plants under drought. In the meantime, it is worth mentioning that the Pn in Pip-pretreated plants was greater than in the untreated drought-affected plants, which is further proof that the chloroplasts experienced less severe injury with Pip pretreatment. Our results were validated by Kucukkalyon and Seckin Dinler [13] under salinity stress on barley seedlings for Pip, as well as for the Pip-like substances SA [27] and NO [28]. Additionally, the Pip application under drought conditions induced the accumulation of carotenoids (Figure 4B), anthocyanins (Figure 9C), and flavonoids (Figure 9D) that are desirable for chlorophyll protection from ROS and maintaining their antioxidant capacity (Figure 9). An additional reason for the increasing chlorophyll concentration following Pip pretreatment is the enhancement of endogenous SA levels (Figure 6D), which is decisive for elevating 5-ALA and chlorophyll [29]. Moreover, Pip pretreatment might regulate the uptake of Fe, Mg, and N, along with the expression of chlorophyll assimilation genes. These results propose that Pip pretreatment boosts the synthesis of chlorophyll molecules under drought by elevating the porphyrin levels and reducing the rate of chlorophyll degradation. These conclusions were validated by Farouk and Al-Ghamdi [8] for Pip-like substances (NO).

Photosynthetic effectiveness relies not only on their genetic potential but also on environmental conditions and agricultural practices [1]. The current study showed that photosynthetic performance criteria (Pn, CA, Rubisco), along with the total carbohydrate concentration, declined by drought, while Pip pretreatment markedly improved the photosynthetic process in barley seedlings under normal or drought conditions (Figure 5). Commonly, photosynthetic processes and Pn were impaired by drought, affecting both stomatal and non-stomatal constraints [5,6]. Stomatal closure in drought-affected plants is linked with the lowest CO_2_ uptake, diminished photosynthetic enzyme activities (CA, Rubisco, and ribulose-1,5-bisphosphate ‘RuBp’), and expression of RuBP regeneration (ribulose-5 phosphate kinase); raising excitation energy, which subsequently induces photodamage and photorespiration, lessening Pn and ultimately photosynthetic efficiency [5]. The current findings revealed that Pip pretreatment through the root medium improved the Pn under normal or drought stress circumstances. This enhancement is attributed to chlorophyll assimilation improvement and lower ROS levels, which protect the photosynthetic system and boost photosynthesis. This increase from Pip pretreatment was associated with the acceleration of SA accumulation in plant tissue (Figure 6D), which plays an important role in the photosynthetic rate. Additionally, as indicated by the current study and a previous study [30], pretreatment with Pip, SA, and NO boosted photosynthetic efficiency by enhancing the activities of regulating enzymes for CO_2_ assimilation, like CA, Rubisco, Rubisco activase phosphoenol pyruvate carboxylase, and diverse transcriptional factors involved in photosynthesis. Additionally, SA may enhance the stability and functionality of the PSII reaction center via upregulating the expression of D1, D2, and LHC proteins, thereby affecting the oxygen-evolving complex [31].

Rubisco is a crucial enzyme involved in the first step of carbon fixation in the photosynthesis of C_3_ plants and is considered the most prevalent enzyme on Earth [32]. Improving Rubisco’s properties and regulation could result in greater photosynthetic efficiency in plants, and several initiatives have been undertaken to modify Rubisco [33]. Due to its importance, numerous researchers have examined Rubisco activity in stressful conditions. In drought-affected plants, Rubisco activity may be hindered depending on the plant’s genotype, water status, CO_2_ concentration, temperature, stomatal restrictions, and the mode of CO_2_ fixation [34]. Alternatively, no studies before recorded the role of Pip treatment on Rubisco activity.

Carbonic anhydrase is a key enzyme involved in photosynthetic carbon metabolism, transport across biological biomembranes, regulating ion exchange, maintaining acid-base homeostasis, and facilitating carbon anabolic processes [35]. The low activity of CA under drought (Figure 5A) indirectly hinders photosynthetic yield through lowering CO_2_ availability to Rubisco [35]. Conversely, Pip pretreatment activates CA, as well as exhibiting excellent flexibility and enhanced regulatory ability in water scarcity. The activity of CA might be on the upsurge because of the wide aperture of stomata, which facilitates a greater uptake of CO_2_. Hence, CA favored the binding of additional CO_2_ molecules with Rubisco, leading to the enhancement and up-regulation of the Calvin cycle and photoassimilate production [36].

Osmotic adjustment has been established as a physiological adaptation strategy alongside drought tolerance [10]. OA in drought-affected barley genotypes revealed that genotypes with high OA capacities preserve cell turgor pressure and plant water status alongside higher productivity, while low OA capacity genotypes had lower yields [5]. This encompasses the hyperaccumulation of OEM. OEMs are highly soluble molecules, non-toxic at molar concentrations, and involved in several metabolic pathways related to enhancing water influx and maintaining cell turgor and OA capacity [37]. The current study validated this theory, which suggested that the superior performance of barley seedlings under drought, owing to Pip pretreatment, may result from a better water status and superior OA capacity (Figure 7) connected with the hyper accumulation of OEMs (Figure 6). Accordingly, the cell Ψs is lessened (more negative), sequentially preserving cellular water homeostasis and enhancing the cell’s ability to retain turgor pressure, desire for better water retention, cell development, stomatal performance, CO_2_ fixation, and photosynthesis, as well as stabilizing macromolecules and ultimately raising drought tolerance [10]. Currently, Pip pre-treatment positively influenced barely seedling water status under normal or drought conditions. It remains unclear how Pip helps in sustaining water status under stressful circumstances. Nonetheless, the current study shows that Pip treatment notably enhanced the buildup of OEM correlated with a reduction in osmotic potential (more negative) and boosted the capacity for osmotic adjustment by increasing water absorption and retention ability (Figure 6). Additionally, the Pip treatment may maintain the water status in plants by inducing stomatal closure and reducing the transpiration rate under biotic stress [38].

RWC acts as an imperative sign of water status in drought-affected barley seedlings, unlike leaf water potential [5]. The reduction in RWC might result from alterations in cellular membrane function and penetrability, as well as their sustainability [39]. The preservation of eminent water relations, chiefly under drought, is crucial for plant species. The current study revealed that Pip pretreatment provided greater RWC than untreated plants (Figure 7A), designating that Pip pretreatment plays an imperative role in supporting superior water status and enhancing drought tolerance in barley seedlings (Figure 1). However, there is a lack of studies regarding Pip’s main role in regulating plant water status. The rise in RWC and water retention capacity by Pip in drought-affected plants might be associated with (1) stomatal closure triggered by elevated concentration of endogenous SA in plant tissue (Figure 6D), associated with a reduction in the transpiration rate, and markedly increased RWC [27]; (2) improved root system development (Figure 1); and (3) an over-accumulation of OEMs (Figure 6), which lowers the osmotic potential (Figure 8E) while boosting the plant’s ability to absorb and retain water and elevated RWC.

Total free amino acid accumulation in drought-affected plants with or without Pip pre-treatment was found to be higher than that in the nontreated control plants (Figure 6A). Amino acids play a crucial role in plant metabolism, serving as the primary product of inorganic nitrogen assimilation, precursors of protein and nucleic acid, and improving drought tolerance through OA capability [40]. This rise might be attributed to the degradation of proteins resulting from ROS, which hinders protein assimilation, and it may occur in response to changes in the OA of their cellular components [40]. Proline is a familiar osmoprotectant that serves as a signal to stabilize biomembranes and proteins, as well as manipulate cell proliferation and stress response gene expression, which are vital for plant revival under stressful conditions [5,6]. Improvement in Pro accumulation within drought and/or Pip pretreatment is linked to protein hydrolysis and a rise in proline biosynthesis precursor, an elevation of ornithine-σ-amino transferase and glutamyl kinase activities, a reduction in proline oxidase, and an increase in ammonia concentration [41]. It is noteworthy to note that Pip-like substance application has been shown to promote Pro accumulation under stress conditions for well-watered control plants. In this concern, with the application of Pip-related molecules, SA [42], and NO [28], it was reported that proline accumulation may be caused by the activation of proline assimilation enzymes and conversion of glutamic-γ-semi-aldehyde into pyrroline-5-carboxylate in both the cytosol and chloroplasts. However, the specific role of Pip in Pro accumulation is not widely recognized and requires further investigation. It is worth mentioning that the excessive accumulation of Pro is consistently associated with a decrease in growth, as its biosynthesis is very energy-consuming [43], which negatively impacts the energy balance needed for growth. This aligns with current outcomes, which show that moderate proline accumulation under Pip pretreatment keeps the cell’s osmotic pressure at an adequate level, facilitating the energy supply necessary for seedling growth under stressful conditions.

Our findings indicate that drought and/or Pip increased SS accumulation in barley seedlings (Figure 6C). Soluble sugars occupy an important role in preserving cell homeostasis, OA, ensuring turgidity and stability of biomolecules and biomembranes, and providing energy and carbons for organic molecule synthesis and cell growth [44]. The increase in SS might be a result of heightened starch hydrolysis, synthesis, or decreased conversion to their product, as well as improved sucrose synthesis-related gene expression [45]. Our results disclose that Pip pretreatment sustains sugar metabolism and lessens photosynthetic activity under drought to diminish stress injury. Yet, there is no information in the literature regarding the interactions between Pip and sugar metabolism under stress. This topic warrants advanced biochemical and molecular analysis in the future.

Typically, a steady state of ROS is crucial in metabolic pathways and the regulation of cell division and differentiation. Conversely, under stressful conditions, the hyper-accumulation of ROS can oxidize or destabilize several physio-biochemical and molecular processes, and ultimately, cell and plant death [39]. The current study revealed that drought stress triggered an increased accumulation of O_2_^−^, H_2_O_2_, MDA, and CMP%, along with a decline in MSI (Figure 8). Alternatively, Pip treatment mitigates oxidative injury to plant metabolic processes by lowering the level of O_2_^−^, H_2_O_2_, MDA, and CMP%, associated with a high MSI (Figure 8). Therefore, Pip pre-treatment might serve as an effective practice to protect plants against oxidative stress. The accumulation of ROS biomarkers noted in the current study could be a result of heightened photorespiration, which ensures a partial replenishment of substrates and maintains the carboxylation function of Rubisco [1]. Additionally, drought induced a reduction in mitochondrial electron transport, which was accompanied by increasing proton generation in the intermembrane space and is associated with a rise in mitochondrial inner-membrane potential (m∆ψ), that declined electron flow in the electron transport chain (ETC), along with an acceleration of the mitochondria stagnation status, and triggers electron leakage from ETC and, ultimately, ROS regeneration [46]. The excessive production of ROS speeds up the accumulation of MDA and upsurges biomembrane dysfunction, which subsequently causes an elevation in the CMP% linked to the lowest MSI (Figure 9C,D) [5]. Therefore, in response to drought-induced oxidative bursts, proper regulation of ROS levels is crucial for sustaining cellular functions, influencing the activation of the antioxidant defense system, and determining the degree of stress tolerance. Consistent with the present study, pretreatment with Pip drastically declined the oxidative biomarkers triggered by plant pathogens via modulating ROS accumulation and the expression of defense-related genes [1,5]. Nevertheless, the imperative role of Pip in the ROS-scavenging system under drought has not been previously examined. The current study indicates that Pip improved drought resilience by upregulating the level of non-enzymatic antioxidants that rapidly dispel ROS and act as chain breakers, thereby limiting injuries (Figure 6 and Figure 9) [47]. Moreover, there are interrelationships between Pip and SA production, suggesting that the mitigatory impact of Pip on oxidative burst arises from a hyperaccumulation of SA, which in turn, can restore and protect cellular membranes by reducing membrane lipid peroxidation, thereby averting electrolyte leakages [48]. This result could be attributed to the activation of the Pip signal transduction, which boosts the defense system to cope with stress.

For sustaining a decisive equilibrium between ROS generation and elimination, plants possess a specialized and intricate antioxidant defense system that eliminates dangerous ROS and protects physio-biochemical pathways from oxidative injury [1]. Non-enzymatic antioxidants like ascorbic acid, tocopherols, glutathione, phenols, anthocyanins, and flavonoids are crucial in modulating ROS and managing oxidative bursts [49]. In the current study, drought and/or Pip pretreatment significantly enhanced non-enzymatic antioxidant solutes over control plants as a strategy for counteracting the drastic impacts of ROS on plant physio-biochemical processes. The current outcomes found that drought typically increased the ascorbic acid in plant tissues. Nonetheless, there is a lack of data concerning the role of Pip in ascorbic acid accumulation. Ascorbic acid serves not only as a key antioxidant in plant cells but also supports membrane-bound antioxidants in their role of cellular protection in two ways [50]: (1) the de-epoxidation of violaxanthin, thus allowing for the dissipation of excess excitation energy, and (2) functioning as a secondary antioxidant by maintaining reduced tocopherol and carotenoids or indirectly via the activation of ascorbate peroxidase. Collectively, these findings bolster the perspective that ascorbic acid has a wide-ranging function in defense responses among various cellular components.

Phenolic compounds are important secondary metabolites, acting as a secondary defense mechanism and involved in the ROS scavenging process through their hydrogen-donating potential and diminishing superoxide-driven Fenton reactions [48]. It has been noted that phenolic compounds accumulate under drought stress and/or Pip pretreatment. This is confirmed by Simontacchiet al. [51] and Farouk and Al-Huqail [52], who indicate that the Pip-linked inducers, SA and NO, induce the release of the contents of phenolic compounds under drought stress. Flavonoids and anthocyanins play an essential role in modulating ROS levels, alongside lowering susceptibility to photoinhibition [53]. Flavonoids contribute to powerful antioxidant/antiradical performances by chelating transition metals (Fe^2+^). For instance, quercetin disrupts ROS formation via the Fenton reaction [54]. Although there has been a chief spotlight on the antioxidant attributes, a growing perspective suggests that flavonoids and their in vivo metabolites do not act as conventional hydrogen-donating antioxidants but may influence cellular activities via actions at protein and lipid kinase signaling pathways [55]. The current study established that Pip can advance the antioxidant capacity of barley seedlings through the assimilation of flavonoids and anthocyanins, as confirmed by the Pip-related substances [52]. This conclusion suggested that exogenous Pip pretreatment significantly activated the ROS-scavenging system, including non-enzymatic antioxidants, to sustain a relatively low level of ROS, while also conferring drought stress tolerance.

In the current study, SOD, CAT, POX, and APX activities declined under drought, suggesting they do not play a role in eliminating excess H_2_O_2_. In this regard, we show that the deactivation of antioxidant enzymes is linked with the hyperaccumulation of H_2_O_2_. Contradictory findings have emerged regarding the activity of antioxidant enzymes under drought, which rely on drought duration and severity, alongside the plant species and cultivar. Consequently, several reports revealed that antioxidant enzyme activities escalated under drought conditions [1,39], while other studies supported our findings showing that antioxidant enzyme activities may decline under drought [56]. The reduction in their activity under drought might result from their suppressive effect on the enzyme system or, possibly, from the deactivation of the enzyme-bound heme group. The decline in APX activity is linked to the accumulation of O_2_^−^ (as seen in histochemical trials, Figure 9) that renders cytosolic APX inactive, thereby reducing its activity. Higher GR activity was correlated to enhanced GSH production and improved defense against ROS [57]. Alternatively, pretreating with Pip under drought enhanced the activity of SOD, CAT, APX, and GR over nontreated drought-affected seedlings. These findings suggested that Pip-mediated increases in one or more antioxidant enzymes played a role in boosting the antioxidant capacity, reflecting a correlation with elevated ROS scavenging and diminished oxidative damage. The specific roles of Pip pretreatment in activating antioxidant enzymes under normal or abiotic stress conditions have not been recorded before. In the meantime, certain reports suggest that Pip-like substances, i.e., NO, mitigate the drastic effect of oxidative injuries by regulating cellular redox homeostasis and facilitating the conversion of O_2_^−^ into water and oxygen, while also activating the H_2_O_2_-scavenging enzymes by upregulating the expression of antioxidative encoding genes. Furthermore, Gula et al. [58] observed that Pip supplementation triggered SOD and CAT enzyme activities under abiotic stress. Meanwhile, Kucukkalyon and Seckin Dinler [13] showed that the application of Pip under salinity stress, in most cases, increased SOD enzyme activity.

## 4. Materials and Methods

### 4.1. Plant Materials and Experimental Outline

Pot experiments were performed in the acclimate room of Plant Stress Physiology Lab., Sinop University, Turkey, using a completely randomized block design with five replicates to evaluate the potential role of Pip in inducing the drought tolerance of barley (*Hordeum vulgare* L. cv, Bülbül89 most moderately drought-tolerant cultivars planted in Turkey, sourced from Ankara Field Crops Research Institute) seedlings. Four treatments were used in the experiment: (1) control, irrigated with full-strength Hoagland solution without polyethylene glycol 6000 or Pip, (2) drought induced by 15% polyethylene glycol 6000 in full-strength Hoagland solution, (3) Pip at 30 µM in full-strength Hoagland solution, and (4) Pip+drought, as indicated in Figure 1. Based on our preliminary experiments, we selected the most suitable levels of Pip (we utilized five concentrations of Pip, i.e., 10, 20, 30, 40, and 50 µM, and chose 30 µM, which gave the superior seedling dry weight and greatest chlorophyll concentration) and polyethylene glycol 6000 (we utilized five concentration from PEG6000, i.e., 5, 10, 15, 20, and 50%, and chose 10%, which greatly declined seedling growth and chlorophyll levels).

**Figure 11:**
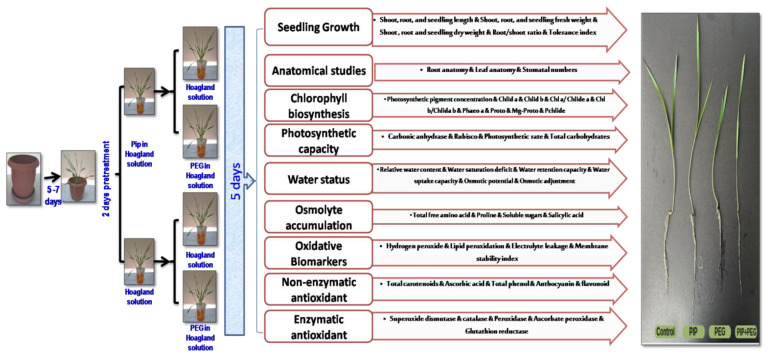
Flowchart of the experimental layout, showing the treatments, date, and studied characteristics.

Each plastic pot (10 × 14 cm) containing peatmoss and vermiculite (1:1 *v*/*v*), five sterilized grains (soaked in 1% sodium hypochlorite solution for 10 min and rinsed with distilled water) was sown and watered until germination and seedling emergence (5–7 days). The uniform seedlings were harvested, cleaned of the germination substrate, and then split into two sets (50 seedlings/set) for Pip pretreatment over 2 days in a glass through the root system. The first set was given a full-strength Hoagland solution, while the second set received 30 μM Pip in a full-strength Hoagland solution. Consequently, each set was divided into two groups (25 seedlings/group) for drought treatment. The first group from each set was grown in full-strength Hoagland solution, whereas the second group from each set received 15% polyethylene glycol 6000 in full-strength Hoagland solution. The four groups were subsequently placed in an acclimated room (16 h light/8 h dark; 60% humidity; 23 °C; and 500 μM/m^2^/s) for five days and then collected for morpho-anatomical and physio-biochemical assessments (Figure 11).

### 4.2. Morphological Characteristics

The seedling shoots and roots were measured along with the whole seedling length, as well as their fresh weight. Subsequently, the seedlings were oven-dried at 70 °C to assess the dry mass of the shoots, roots, and intact seedlings. The tolerance index was calculated by dividing the total dry mass of the treated seedlings by the total dry mass of the control seedlings and was expressed as a percentage [59].

### 4.3. Leaf and Root Anatomy

The root (0.5 cm in length, 3 cm from the root tip) and leaf (0.7 cm from the 1st upper mature leaf) pieces were fixed for 48 h in a fixative solution (37% formalin, acetic acid, 50% alcohol; 5:5:90; *v*/*v*/*v*). The plant specimens were subsequently dehydrated in an ethanol series, embedded in paraffin blocks, and then cross-sectioned (15 µm thickness) with an Accu-Cut^®^SRM™ 200 Rotary Microtome (Sakura Finetek Inc., Street Torrance, CA, USA) and stained with Safranin O/Fast-Green protocol [60]. All sections were examined using an optical microscope DM3 XL (Leica Microsystems, Thermo Fisher Scientific Inc., Waltham, MA, USA) and photographed.

### 4.4. Stomatal Density

Leaf imprints of the adaxial and abaxial epidermis at the central portion of the leaf were prepared via cellulose acetate in acetone [61]. After drying, each imprint was carefully removed from the leaf, mounted on a glass slide, and then examined by light microscope, and the stomatal number was recorded in the microscope’s field area (0.25 mm^2^).

### 4.5. Determination of Chlorophyll Metabolic Intermediates

Chlorophyllide (Chlide) levels were measured using the adapted scheme of Harpaz-Saad et al. [62]. The leaf disks were ground in a prechilled mortar and pestle with acetone and subsequently filtration. The aliquots of the filtrates were transferred into centrifuge tubes that held hexane and KOH, then vortexed and centrifuged for phase separation. The Chl concentration was deliberated in the hexane phase, and Chlide concentrations were appraised in the acetone phase spectrophotometrically (Thermo Scientific Genesys 10S UV-Vis Spectrophotometer, Waltham, MA, USA).

The level of pheophytin_a_ (Pheo_a_) was assayed using the adapted protocol of Radojevič and Bashkin [63]. The leaf disks were ground in a chilled mortar and pestle using acetone containing magnesium carbonate and subsequently filtered. Aliquots of extracts were transferred to cuvettes, and optical density (OD) was recorded at 664 and 750 nm. Next, 0.1 mL of HCl was added and mixed thoroughly. Then, the OD at 665 and 750 nm was recorded, and their concentration was calculated.

The porphyrin concentration was estimated using the scheme outlined by Sarropoulou et al. [64]. The leaf disks were placed into test tubes and mixed with ethanol. The samples were placed in a water bath at 65 °C until discoloration of the samples. Consequently, the concentrations of protoporphyrin (Proto), Mg-protoporphyrin (Mg Proto), and protochlorophyllide (Pchlide) were determined.

Proto (µg g^−1^ FW) = [(12.25 × A665 − 2.55 × A649) × volume of supernatant (mL)/sample weight (g)]/892.

MgProto (µg g^−1^ FW) = [(20.31 × A649 − 4.91 × A665) × volume of supernatant (mL)/sample weight (g)]/906.

Pchlide (mg g^−1^ FW) = [(196.25 × A575 − 46.6 × A590 − 58.68 × A628) + (61.81 × A590 − 23.77 × A575 − 3.55 × A628) + (42.59 × A628 − 34.32 × A575 − 7.25 × A590)] × volume of supernatant (mL)/sample weight (g) × 1000.

### 4.6. Photosynthetic Pigment Concentration

To quantify the chlorophyll (a, b, and total) and carotenoid concentrations, a leaf sample (0.05 g FW) was extracted by soaking in ethanol (10 mL) enriched with sodium bicarbonate (0.5%) till the discoloration of plant tissue at 4 °C [65]. The extract’s optical density was recorded at 470, 648, and 664 nm with a spectrophotometer, and subsequently, their concentrations were calculated using the following equation:

Chlorophyll_a_ (mg g^−1^ FW) = 13.36 A664 − 5.19 A648

Chlorophyll_b_ (mg g^−1^ FW) = 27.34 A648 − 8.12 A664

Carotenoid (mg g^−1^ FW) = (1000 A470 − 2.13 Cchlorophyll a − 97.64 Cchlorophyll b)/209

Chlorophyll stability index (CSI) was deliberate with Sairam et al. [66] formula. CSI = $\frac{Total\;chlorophyll\;under\;stress}{total\;chlorophyll\;under\;control}\times 100$.

### 4.7. Photosynthetic Performance Features

The protocol presented by Dwivedi and Randhawa [67] was utilized to judge CA activity (μmol CO_2_ kg^−1^ leaf FW S^−1^) in fresh leaves. At 4 °C, leaf pieces were immersed in 0.2 M cysteine hydrochloride solution for 20 min. Subsequently, the leaf fragments were transferred to a phosphate buffer (pH 6.8) solution. To this mixture, we added sodium bicarbonate solution and bromothymol blue. Ultimately, the solution was titrated with HCl using methyl red as an indicator. Leaf ribulose 1,5-bisphosphate carboxylase oxygenase was extracted, and its activity was assessed with a modified spectrophotometric technique of Lu et al. [68] at 340 nm. The photosynthetic rate of fully mature leaves was measured using EARS miniPPM Models 200/300 (EARS, Almere, The Netherlands).

Total carbohydrates were extracted and analyzed as explained by Sadasivam and Manickam [69]. Dried plant powder was transferred to a glass centrifuge tube with 1.5 N H_2_SO_4_ and then underwent centrifugation. One milliliter of the extract was taken in a test tube to which 1 mL of 5% phenol was added. The OD was recorded at 490 nm using a spectrophotometer and compared to the calibration curve of glucose, which was established using known concentrations from 0 to 100 μg/mL.

### 4.8. Osmotically Energetic Molecules

The concentration of TAA (mg/g FW) in barley leaves was assessed following the Sadasivam and Manickam [69] procedure with minor modifications regarding ninhydrin reagent spectrophotometry at 570 nm. Glycine served as a standard amino acid to create a calibration curve (0–100 μg/mL) from which the amount of free amino acid was extrapolated. Free Pro (mM proline/g FW) was extracted from fresh leaves using 3% (*w*/*v*) aqueous sulphosalicylic acid and quantified with the ninhydrin reagent [70]. The absorbance of the fraction containing toluene aspirated from the liquid phase was recorded at 520 nm.

Soluble sugar concentrations were estimated using the phenol/sulfuric colorimetric technique [69]. Dried seedling powder was mixed with ethanol in a 10 mL glass tube, heated at 80 °C for 30 min, cooled down, and then centrifuged. Subsequently, 0.1 mL of extract was mixed with a 5 mL phenol–sulfuric acid solution at 90 °C for 15 min. The reaction solution was measured by a spectrophotometer at 620 nm. Salicylic acid was extracted and estimated spectrophotometrically at 540 nm with ferric chloride, as described by Warrier et al. [71].

### 4.9. Water Status and Osmotic Adjustment

The relative water content (RWC), water retention capacity (WTC), water saturation deficit (WSD), and water uptake capacity (WUC) were estimated based on the formula suggested by Islam and Mohammad [72]. The RWC% was assessed using leaf segments that were speedily weighed to attain fresh mass (FM). Later, this was floated on distilled water to rehydrate it and was weighed once again to achieve a turgid mass (TM). The turgid samples were consequently oven-dried to achieve dry mass (DM). RWC% was deliberated using this formula: RWC (%) = [(FM − DM/(TM − DM)] × 100. Additionally, the seedlings’ WSD, WTC, and WUC were calculated using the following formulas: WSD = 100 − RWC; WTC = TM/DM; and WUC = (TM − FM)/DM.

The osmotic potential (Ψs) of barley leaves was assessed by the technique outlined by Janardhan et al. [73] through an electrical conductivity meter. The osmotic adjustment capacity (OA) was anticipated by the difference in osmotic potential among stressed and control plants [74].

### 4.10. Oxidative Biomarkers

The hydrogen peroxide (H_2_O_2_) concentration (μmol g^−1^ FW) in barley leaves was extracted and determined colorimetrically following the procedure outlined by Velikova et al. [75]. Lipid peroxidation (µM MDA g^−1^FW) was appraised by measuring malondialdehyde (MDA) concentration generated via the reaction with thiobarbituric acid, following the Madhava Rao and Stresty [76] protocol. Plant samples were homogenized in 1% trichloroacetic acid (TCA) and centrifuged at 10,000× *g* for 10 min. The supernatant was added to 1 mL0.5% (*w*/*v*) thiobarbituric acid (TBA) in 20% TCA and, then, incubated in boiling water for 30 min. The samples were centrifuged at 10,000× *g* for 5 min, and the supernatant OD was measured at 532 nm. The value for nonspecific OD at 600 nm was subtracted.

Cellular membrane permeability (CMP) was evaluated using the Singh et al. [77] procedure with slight modifications. The leaf samples were soaked in 15 mL of distilled water for 6 h, and then, the electrical conductivity (EC1) was measured with a conductivity meter (Hanna Instruments, Buzzard, UK). The samples were boiled for 15 min., cooled at room temperature, and subsequently, the conductivity was re-measured (EC2). The CMP% was estimated using the following formula: CMP (%) = (EC1/EC2) × 100. The membrane stability index (MSI) was anticipated by Sairam et al. [66]. Fresh leaves were immersed in 15 cm^3^ of double-distilled water in two sets. One set was subjected to 40 °C for 30 min, and its conductivity was recorded by an electric conductivity meter (C1). The second set was kept in a boiling water bath for 10 min., and its conductivity was also taken (C2). MSI= $\left\lfloor 1-(\frac{C1}{C2})\right\rfloor \times 100$.

### 4.11. In Situ Hydrogen Peroxide and Superoxide Anions Localization

Histochemical staining techniques employing nitroblue tetrazolium (NBT) and 3,3′-diaminobenzidine (DAB) were utilized to assess the accumulation of peroxide (O_2_^−^) and H_2_O_2_ in leaves, respectively [78]. For NBT or DAB staining, the samples were immersed in 1 mg/mL NBT or DAB solution prepared in phosphate buffer (pH 7.8) at the lab temperature under light. When blue (NBT staining) or brown (DAB staining) spots emerged, the stained samples underwent clearing in concentrated ethanol and were stored in 70% ethanol before being photographed.

### 4.12. Non-Enzymatic Antioxidant Metabolites

Oxalic acid was used to extract ascorbic acid, followed by titration with 2,6-dichlorophenol indophenols, following the Sadasivam and Manickam [69] method. The concentration of soluble phenols (mg gallic acid g^−1^FW) was assessed following the method established by Sadasivam and Manickam [69]. Sample extracts were reacted with sodium bicarbonate, and Folin–Ciocalteau reagent was consistently heated for 30 min at 40 °C. The OD of a blue complex was recorded at 650 nm with a UV–visible spectrophotometer.

The anthocyanin concentration (μgg^−1^FW) was determined, as outlined by Rabino and Mancinelli [79]. Fresh seedling (1 g) was extracted with acidified methanol (1% HCl) at 0 °C for 72 h. The mixture was subsequently centrifuged, and the supernatant OD was recorded at 530 nm and 657 nm. The flavonoids were determined to be consistent with the scheme of Lin et al. [80] with 80% ethanol containing 1% HCl as solvent. Sampling of the supernatants was taken to appraise the OD at 540 nm and subsequently expressed as A540/g FW.

### 4.13. Antioxidant Enzymes Assay

Frozen leaves (0.5 g) were ground in liquid nitrogen, and the resulting powder was suspended in an extraction buffer (potassium phosphate, pH 7.0, and Na 2-ethylenediaminetetra acetic acid (EDTA), having 5% (*w*/*v*) polyvinylpolypyrrolidone (PVP). The homogenates were centrifuged, and the supernatant fraction was utilized for enzyme activities (unit mg^−1^ protein) and protein concentration. The protein concentration in the extracts was measured following Bradford [81], utilizing bovine serum albumin as a standard.

Superoxide dismutase (SOD, EC1.15.1.1) activity was assessed with NBT [82]. The reaction mixture contained Na-phosphate buffer, pH 7.3, methionine, NBT, EDTA, riboflavin, and enzyme extract. The reaction was initiated by adding riboflavin, and the glass test tubes were shaken and positioned beneath fluorescent lamps (60 mmol m^2^ s^1^). The reaction was permitted to continue for 5 min and was subsequently stopped by switching off the light. The OD was recorded at 560 nm. Blanks and controls were performed similarly but without illumination and enzyme, respectively. One unit of SOD was defined as the quantity of enzyme that produced 50% inhibition of NBT reduction under testing conditions. Catalase (CAT, EC 1.11.1.7) activity was assessed by measuring the initial rate of H_2_O_2_ depletion utilizing the Wang and Huang [82] protocol. Briefly, the 3 mL reaction mixture included phosphate buffer (pH 7.0), H_2_O_2_, and enzyme extract. The decomposition of H_2_O_2_ was assessed at 240 nm. An extinction coefficient of 39.4 mM^−1^ cm^−1^ was used for the estimated CAT activity (1 unit = 1 mM of H_2_O_2_ reduction/min). The method of Nakano and Asada [83] was employed to determine the ascorbate peroxidase (APX, EC 1.11.1.11) activity. The assay mixture contained phosphate buffer (pH 7.0), EDTA, ascorbate, H_2_O_2_, and enzyme extract. The decline in the OD of ascorbate at 290 nm was recorded. The activity was deliberate via the extinction coefficient 2.8 mM^−1^ cm^−1^. Glutathione reductase (EC 1.6.4.2) was assessed by the Foyer and Halliwell [84] method. The assay mixture contained phosphate buffer pH 7.8, GSSG, NADPH-Na_4_, and extract. NADPH oxidation was determined at 340 nm. Calculation was conducted via the extinction coefficient of glutathione reduction enzymes (6.2 mm^−1^ cm^−1^). One unit of GR was defined as 1 mmol mL^−1^ GSSG reduced min^−1^.

### 4.14. Statistical Analysis

One-way analysis of variance (ANOVA) was carried out, and after that, Tukey’s multiple range test between the means of treatments was used to determine the significant differences (*p* ≤ 0.05) between the mean values. All values described are the mean of five replicates with ± standard error (SE). The data were analyzed using CoHort Software, 2008 statistical package (CoHort Software, 2006; release 6.3.0.3, 2006 CoStat Institute, Cary, NC, USA).

## 5. Conclusions

The current investigation highlighted that Pip pretreatment at 30 μM via a rooting medium lessens the detrimental effects of drought on barley seedlings by boosting chlorophyll biosynthesis, optimizing plant water status and preserving the osmotic adjustment capacity, maintaining redox homeostasis, and potentially improving barley seedling drought tolerance. This information creates opportunities for innovative approaches aimed at improving drought tolerance in crops. Future research endeavors might explore deeper into elucidating the precise molecular mechanisms underlying Pip action and its impact on nutrient uptake to improve plant drought tolerance, and a field-scale experiment is needed.

## Figures and Tables

**Table 1:** Effect of drought stress and pipecolic acid and their interaction on root and leaf anatomical features and stomatal density of the abaxial and adaxial surface of barley seedlings.

Treatment	Root				Leaf						Stomatal Density	
	Epidermis Thickness (µm)	Cortex Thickness (µm)	Stele Diameter (µm)	Thickness of Vascular Tissues	Leaf Blade Thickness (µm)	Mesophyll Tissue Thickness (µm)	Thickness of Leaf at Midrib Region (µm)	Main Vascular Bundle Dimension (µm)		Metaxylem Vessel Diameter (µm)		
								Length	Width		Adaxial	Abaxial
Control	18.75 ± 0.03 a	137.5 ± 0.33 b	131.2 ± 2.32 b	80.25 ± 1.33 b	143.7 ± 1.04 c	118.7 ± 1.15 c	212.5 ± 0.40 c	56.25 ± 0.60 b	87.50 ± 0.53 a	6.87 ± 0.29 b	72.66 ± 0.44 bc	64.33 ± 2.66 bc
Pipecolic acid	18.75 ± 0.23 a	193.7 ± 0.33 a	150.0 ± 0.46 a	99.43 ± 1.26 a	193.7 ± 2.42 a	150.0 ± 0.55 a	318.7 ± 1.60 a	68.75 ± 0.52 a	75.00 ± 0.46 b	10.0 ± 0.49 a	67.83 ± 1.09 c	57.00 ± 1.04 c
Drought	6.250 ± 0.11 b	84.30 ± 1.10 d	80.25 ± 0.02 d	45.22 ± 0.39 d	137.5 ± 0.80 c	106.2 ± 1.75 d	200.0 ± 0.49 d	56.25 ± 0.22 b	75.00 ±0.5 b	5.70 ± 0.29 b	102.0 ± 1.52 a	74.50 ± 1.80 a
Drought + Pipecolic	18.75 ± 0.81 a	100.0 ± 1.48 c	87.50 ± 1.09 c	60.17 ± 0.22 c	162.5 ± 0.40 b	131.2 ± 0.60 b	243.7 ± 2.17 b	56.25 ± 0.13 b	75.00 ± 0.24 b	6.30 ± 0.12 b	85.60 ± 6.25 b	68.66 ± 2.84 ab
*p* value	***	***	***	***	***	***	***	***	***	***	***	**

Data represented as the mean ± standard error (*n* = 5). Significant differences between groups were tested by one-way analysis of variance (ANOVA, *p* ≤ 0.05). Data of each column indicated by the same letters are not significantly different (*p* ≤ 0.05), as estimated from Tukey’s test. Significance levels are indicated by *** *p* ≤ 0.001 and ** *p* ≤ 0.001.

## Data Availability

No data was used for the research described in the article.

## Supplementary Materials

The following supporting information can be downloaded at: https://www.mdpi.com/article/10.3390/plants14131949/s1.
