# Supplementary material for: Pipecolic Acid, a Drought Stress Modulator, Boosts Chlorophyll Assimilation, Photosynthetic Performance, Redox Homeostasis, and Osmotic Adjustment of Drought-Affected Hordeum vulgare L. Seedlings
Source: Plants (Basel). 2025 Jun 25;14(13):1949. doi: 10.3390/plants14131949 (PMC12252321; doi:10.3390/plants14131949)
Supplement: Supplementary file 1 [file plants-14-01949-s001.zip › plants-3703654-supplementary.pdf]

Supp. 1

Overview of one-way analysis of variances ANOVA concerning the impact of drought and/or pipecolic acid on certain morpho-physiological and biochemical traits of barley seedlings

Characteristics	Degree of freedom (df)	Sum of square (ss)	Mean square (Ms)	F-value	P-value
Root length	3	48.190	16.060	9.4160	0.0053**
Shoot length	3	124.07	41.350	21.500	0.0003***
Seedling length	3	317.82	105.94	17.190	0.0008***
Root fresh weight	3	0.0122	0.0040	5.8330	0.0206*
Shoot fresh weight	3	0.0884	0.0294	39.550	0.0000***
Seedling fresh weight	3	0.1660	0.0553	26.820	0.0002***
Root dry weight	3	0.0001	0.0001	15.300	0.0011**
Shoot dry weight	3	0.0001	0.0001	4.1370	0.0480*
Seedling dry weight	3	0.0019	0.0001	15.460	0.0011**
Shoot tolerance index	3	2707.6	902.55	11.970	0.0025**
Root epidermis thickness	3	351.56	117.18	213.92	0.0000 ***
Root cortex thickness	3	22114	7371.5	2691.1	0.0000 ***
Root stele diameter	3	10267	3422.6	670.57	0.0000 ***
Root thickness of vascular tissues	3	5026.3	1675.4	634.01	0.0000 ***
Leaf blade thickness	3	5742.1	1914.0	328.06	0.0000 ***
Leaf mesophyll tissue thickness	3	3134.7	1044.9	274.22	0.0000 ***
Thickness of leaf at midrib region	3	25546	8515.6	1473.7	0.0000 ***
Leaf main vascular bundle dimension length	3	351.56	117.18	217.26	0.0000 ***
Leaf main vascular bundle dimension width	3	334.43	111.47	179.52	0.0000 ***
Leaf metaxylem vessel diameter	3	34.244	11.414	35.280	0.0001 ***
Stomatal density at Adaxial surface	3	2123.4	707.80	22.047	0.0003 ***
Stomatal density at Abaxial surface	3	489.22	163.07	11.118	0.0032 **
$Chlorophyllide_a$	3	0.5942	0.1980	5.6935	0.0220 *
$Chlorophyllide_b$	3	1.4090	0.4696	8.4770	0.0073 **
$Chlorophyll_a$ (Chl_a)/ $Chlorophyllide_a$	3	23.148	7.7162	7.8990	0.0089 **
$Chlorophyll_b$ (Chl_b)/ $Chlorophyllide_b$	3	21.830	7.2768	13.976	0.0015 **
$Pheophytin_a$	3	0.0367	0.0122	54.074	0.0000 ***
Protoporphyrin	3	0.0373	0.0124	55.500	0.0000 ***
Magnesium-protoporphyrin	3	0.5879	0.1959	5.8996	0.0200 *
Protochlorophyllide	3	0.1101	0.0367	44.480	0.0000 ***
$Chlorophyll_a$	3	0.7566	0.2522	27.449	0.0001 ***
$Chlorophyll_b$	3	0.6701	0.2233	61.012	0.0000 ***
Total chlorophyll	3	2.8417	0.9472	44.139	0.0000 ***
Carotenoid	3	0.6713	0.2237	73.884	0.0000 ***
Chlorophyll stability index	3	6131.3	2043.7	17.597	0.0007 ***
Carbonic anhydrase activity	3	52483	17494	16.812	0.0008 ***
Rubisco activity	3	0.0170	0.0056	0.2194	0.0082*
Photosynthetic rate	3	500.32	166.77	24.522	0.0002 ***
Total carbohydrates	3	39041	13013	50.284	0.0000 ***
Total amino acid	3	324.20	108.06	41.778	0.0000 ***
Proline	3	59.829	19.943	201.33	0.0000 ***
Soluble sugar	3	59.830	19.943	201.53	0.0000 ***
Salicylic acid	3	57.302	19.100	99.439	0.0000 ***
Relative water content	3	453.73	151.24	57.919	0.0000***
Water saturation deficient	3	453.88	151.29	58.136	0.0000***

Water retention capacity	3	25.961	8.6533	4.1068	0.0489*
Water uptake capacity	3	3.1629	1.0543	15.936	0.0010***
Osmotic potential	3	0.6994	0.2331	19.489	0.0005 ***
Osmotic adjustment	3	0.6994	0.2331	16.516	0.0009 ***
Hydrogen peroxide	3	153.16	51.056	63.323	0.0000 ***
Malondialdehyde	3	0.0045	0.0015	150.46	0.0000 ***
Cellular membrane permeability	3	914.02	304.67	16.014	0.0010***
Membrane stability index	3	913.69	304.56	304.56	0.0010***
Ascorbic acid	3	195.46	65.154	83.316	0.0000***
Phenol	3	0.0367	0.0122	53.223	0.0000 ***
Anthocyanin	3	1548.0	516.03	7.5087	0.0103 *
Flavonoid	3	11.760	3.9200	4.0692	0.0499 *
Superoxide dismutase	3	0.7501	0.2500	413.49	0.0000 ***
Catalase	3	0.0122	0.0040	9.1310	0.0058 **
Peroxidase	3	0.1966	0.0655	5.6190	0.0227 *
Ascorbate peroxidase	3	0.1133	0.0779	9.4576	0.0052 **
Glutathione reductase	3	1.1241	0.3747	1.0095	0.0004374*

Numbers represent F-values: *** P \0.0001, ** P\0.001, * P \0.05, ns non-significant
